# Alkaloids as Photosensitisers for the Inactivation of Bacteria

**DOI:** 10.3390/antibiotics10121505

**Published:** 2021-12-08

**Authors:** Sònia López-Molina, Cristina Galiana-Roselló, Carolina Galiana, Ariadna Gil-Martínez, Stephane Bandeira, Jorge González-García

**Affiliations:** 1 Department of Inorganic Chemistry, Institute of Molecular Science, Catedrático José Beltran 2, 46980 Paterna, Spain; sonia.lopez@uv.es (S.L.-M.); cristina.galiana@uv.es (C.G.-R.); ariadna.gil@uv.es (A.G.-M.); bandeira.phane@gmail.com (S.B.); 2 Department of Pharmacy, CEU Cardenal Herrera University, Ramón y Cajal s/n, 46115 Alfara del Patriarca, Spain; carol@uchceu.es

**Keywords:** alkaloid, photosensitiser, photoinactivation of bacteria, photomicrobial

## Abstract

Antimicrobial photodynamic therapy has emerged as a powerful approach to tackle microbial infections. Photodynamic therapy utilises a photosensitiser, light, and oxygen to generate singlet oxygen and/or reactive oxygen species in an irradiated tissue spot, which subsequently react with nearby biomolecules and destroy the cellular environment. Due to the possibility to irradiate in a very precise location, it can be used to eradicate bacteria, fungus, and parasites upon light activation of the photosensitiser. In this regard, natural products are low-cost molecules capable of being obtained in large quantities, and some of them can be used as photosensitisers. Alkaloids are the largest family among natural products and include molecules with a basic nature and aromatic rings. For this study, we collected the naturally occurring alkaloids used to treat microorganism infections using a photodynamic inactivation approach. We gathered their main photophysical properties (excitation/emission wavelengths, quantum yields, and oxygen quantum yield) which characterise the ability to efficiently photosensitise. In addition, we described the antibacterial activity of alkaloids upon irradiation and the mechanisms involved in the microorganism killing. This review will serve as a reference source to obtain the main information on alkaloids used in antimicrobial photodynamic therapy.

## 1. Introduction

The spread of resistant parasites, fungi, and bacterial strains is one of the main threats to humanity as recognised by the World Health Organisation (WHO) in its report in 2014 [1] and its priority list of pathogens published in 2017 [2]. Currently, bacterial infections are treated with antibiotics developed in the 1980s and 1990s, and the pipeline of new drugs has diminished in recent years. The widespread use of these antibiotics during the last 30 years has resulted in less effective, or even ineffective, clinical treatments. We have come so far because microorganisms can natively be resistant to antimicrobials, develop antimicrobial resistance via genetic mutations, or acquire antimicrobial-resistant phenotypes from the transmission of adjacent colonies [3]. Furthermore, the pharma industry has systematically ignored antimicrobial development for microorganism infections because of the low economic profit in comparison with other clinical treatments [4,5]. The microbial infections have arisen in recent years and are estimated to cause an annual death toll of 10 million worldwide by 2060, making many of the medical advances of the 20th century obsolete [6,7]. In this line, drugs with novel mechanisms of action and/or new approaches are needed to overcome this global health issue.

Alkaloids are secondary plant metabolites that have been used traditionally in a large variety of medical issues, ranging from cancer and neurodegeneration to bacterial and parasitic infections [8]. They are molecules containing heteroaromatic rings with at least one nitrogen in the ring system, giving them a basic nature. Alkaloids show diverse and interesting photophysical properties, such as long excitation/emission wavelengths and high quantum yield. In view of the importance of alkaloids as traditional chemotherapeutics and the increased demand in developing new approaches to tackle microbial-resistant infections, there has been a recent interest to apply alkaloids for the photoinactivation of microbials.

This review seeks to integrate the new therapeutic applications of alkaloids in antimicrobial photodynamic inactivation to date. Through this approach, alkaloids can potentially kill microorganisms by either the mechanism of action of the molecule or by the reactive species generated under irradiation or a combination of both of them. This opens the wave for enhancing the antimicrobial activity and the application of this approach for clinical treatments in the near future.

## 2. Overview of Alkaloids in the 21st Century

Alkaloids are one of the largest family of natural products with a diverse range of medical applications, including analgesic, central nervous stimulant/depressant, anticholinergic, antitumour, and antiparasitic activities [9,10,11,12]. They have been used in traditional medicine for centuries because of their presence in bacteria, fungi, plants, amphibians, insects, mammals, and humans; more than 18,000 alkaloids have been discovered thus far [13].

Carl Friedrich Wilhelm Meissner first coined the term alkaloid in 1818, to describe substances that had alkaline properties, and the name literally means alkali-like [14]. Alkaloids show a wide structural diversity which is characterised by, at least, a single basic nitrogen atom within the structure. This nitrogen atom can exist as a primary, secondary, and tertiary amine with basic properties, which have been exploited for extraction and purification. The acid-base properties change depending on the global structure and the functional groups. As free bases, alkaloids are soluble in organic solvents and non-soluble in aqueous solution, although the protonation of the amine to form the acid form solubilises the alkaloid in water and insolubilises in apolar solvents [8].

The most common classification of alkaloids occurs attending to the chemical structure in (i) heterocyclic alkaloids or (ii) non-heterocyclic alkaloids or protoalkaloids, in which the nitrogen is located in the heterocyclic moiety or the side chain, respectively. Other manners to classify alkaloids are also possible such as the natural or biochemical origin. They are termed in many different ways recalling the originating organism, geographic location of the source, the pharmacological activity, or the discoverer but ending in ‘-ine’ [15].

Alkaloids are, at present, an interesting group for medicinal chemicals because of the large number of new molecules yet to be discovered from unexplored species and the broad range of applications that enable some drugs to be repurposed for other diseases [16,17,18,19]. In this line, chloroquine has attracted much attention due to its potential applications for antiviral treatment. Of utmost importance, old alkaloids can nowadays be combined with drug delivery technologies such as liposomal and nanomaterial systems to overcome issues such as targeting a particular location or controlling drug release in cancer or microbial infections [20,21]. 

## 3. Antimicrobial Photodynamic Therapy

Photodynamic therapy (PDT) has been used in different applications, although cancer has attracted most of the efforts during the last few decades. Traditional cancer treatments include immunotherapy, chemotherapy, and radiotherapy but are associated with side effects and drug resistance; thus, PDT has emerged as a feasible complementary technique for cancer. Currently, PDT has arisen passed from a promising modality to clinical use to treat several types of cancer in the last two decades, including breast, oral cavity, head and neck, skin, oesophageal, and bladder cancer [22,23,24]. One example of a drug as a photosensitiser for photodynamic therapy is Rostaprfin (Purlytin^®^) which is currently been investigated in phase II/III clinical trials for the treatments of metastatic breast cancer [23]. Nevertheless, other applications of PDT are poorly explored but remain with a bright future such as ophthalmology [25,26], urology [27], and antimicrobial infections [28]. In this line, only a few clinical investigations using antimicrobial photodynamic therapy (aPDT) have been reported, such as the use of α-aminolaevulinic acid for the treatment of chronic skin ulcers in lower limbs infected with *P. aeruginosa* [29] and the application of phenothiazinium dyes for the treatment of candidiasis infections [30].

Despite being the microorganism paramecia, one of the first models reported to be inactivated with photosensitisers and light more than 100 years ago [31], only recently, the application of PDT to kill microorganisms have arisen the interest of the scientific community [32]. This emerging interest in PDT arises from the decrease in the antimicrobial effect of current drugs and the apparition and dissemination of superbugs capable to resist any antibiotic treatment [33,34]. The application of photodynamic processes to kill microbials has been granted several terms in the last decade, including photodynamic inactivation of bacteria (PDI), photodynamic antimicrobial chemotherapy (PACT), or antimicrobial photodynamic therapy (aPDT) [35].

**Figure 1:**
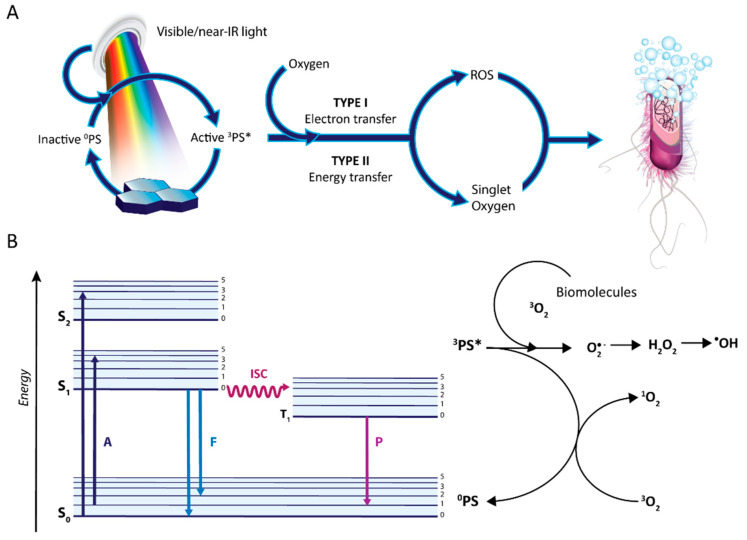
(**A**) Schematic representation of aPDT process; (**B**) representation of the PDT process with a Jablonski diagram.

PDT relies on the absorption of photons at a particular wavelength by molecules termed photosensitisers (PSs), which causes the excitation of one electron from the ground state (HOMO) into a higher energy orbital (LUMO) (Figure 1) [36].

The PS in the excited singlet state decays (S_1_) to the ground singlet state (S_0_), releasing energy either as fluorescence emission or as heat (internal conversion). Nevertheless, the PS in the S_1_ may undergo an intersystem crossing process to form an excited triplet state (^3^PS*, see Figure 1). From this ^3^PS*, the photosensitiser can be deactivated to the ground singlet state by phosphorescence and via two mechanisms of action associated with PDT and termed as type I and II [37].

Type I is a complex mechanism yielding several reactive intermediates and generating reactive oxygen species (ROS), which are reactive chemicals formed from molecular oxygen(^3^O_2_). These ROS include superoxide anion (O_2_^−^), hydrogen peroxide (H_2_O_2_), and hydroxyl radical (HO^∙^) formed via the Fenton reaction. In the type II mechanism, the PS excited triplet state (^3^PS*) transfers energy to ^3^O_2_ and generates highly reactive singlet oxygen (^1^O_2_) species. All of these species can effectively photooxidise a wide range of biomolecules such as proteins and lipids causing the lysis of cell membranes and substantial biological damage. Of utmost importance, PS can undergo both types of mechanisms simultaneously. Some natural products such as anthraquinones are photosensitisers acting through both types of mechanisms [38].

The advantages of aPDT include (i) the low systemic toxicity, (ii) minimal invasiveness, (iii) lack of initiating resistance, (iv) the low mutagenic potential, (v) the efficient inactivation of antibiotic-resistant strains, (vi) a broad spectrum of action, and (vii) the lack of producing photoresistant microbial cells upon multiple treatments. The key characteristics of an adequate PS in order to show high antimicrobial efficacy comprise (i) low toxicity towards mammalian cells, including in the dark; (ii) high ^1^O_2_ quantum yield (Φ_Δ_); (iii) photostable; (iv) broad spectrum of antimicrobial action (bacteria, fungi, parasites); (v) high binding affinity for microorganisms; (vi) no mutagenicity; (vii) high aqueous solubility [39,40,41].

The existence of underlying differences between PDT in cancer and aPDT for microbial infections must be taken into account because of the different mechanisms used to reduce the oxidative stress from the environment between humans and bacteria. Antioxidant molecules in bacteria include carotenoids, while photosynthetic active organisms have chlorophylls, which are different from human reducing agents (thiols, ascorbic acid, etc.). As antioxidative protection, a wide variety of proteins detoxify ROS and regulate redox homeostasis such as superoxide dismutase, catalase, and peroxidase, which have a strong effect in iron-based reactions such as Fenton reaction in photodynamic processes [42].

Apart from the type and amount of ROS generated, another interesting factor is the localisation of the PS because ROS react and eliminate the direct surrounding biomolecules. Even if the PS is only attached to the bacterial surface/cell wall area, oxidation of molecules occurs only at this site, leading to loss of function of proteins, enzymes, and fatty acids [28,39]. Even when the PS is attached only to the surface of bacteria, the intracellular localised defence systems cannot assist bacteria to survive the excess of oxidative burst induced by aPDT. As a consequence, aPDT is potentially a very fast and effective approach to inactivate antibiotic-sensitive bacteria as well as multiresistant strains.

It is worthy to mention the bacterial biofilms since their tolerance against antimicrobials is much higher. Thus far, the efficacy of many PS has been already proved for the inactivation of biofilm growth although bacteria can still secrete exogenous or endogenous pigments in the biofilm that may act as ROS scavengers and reduce aPDT susceptibility [43,44].

Another key parameter for the application of aPDT is the scarce dosimetry of light to the PS, and thus, new irradiation sources have been developed to improve the penetration depth and control the doses to the target site. In the beginning, conventional arc lamps were used because they are safe, easy to use, and inexpensive. Currently, lasers are widely used to yield a high-energy monochromatic light of a specific wavelength with a narrow bandwidth for a specific PS. As infection locations can be found deep in the body, the tissue penetration of the light has to be considered; blue irradiation allows penetration of 1 mm, while irradiation towards the near-infrared region increases the penetration (i.e., red irradiation 0.5–1 cm).

## 4. Alkaloids in Photoinactivation of Microorganisms

**Scheme 1:**
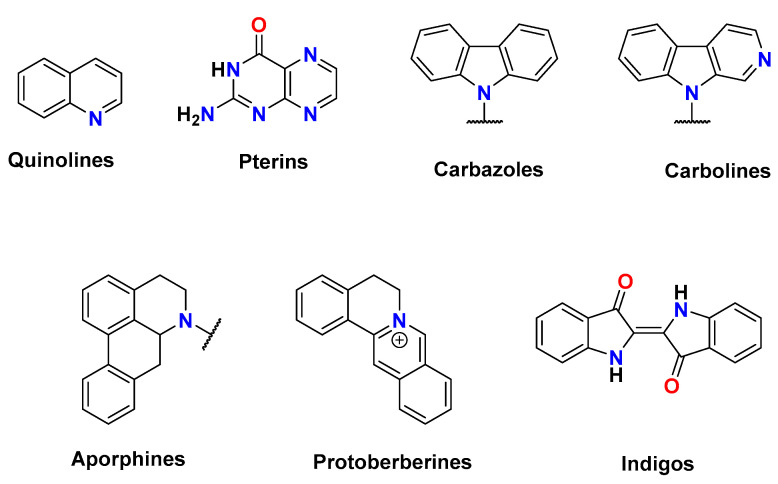
Families of alkaloids of this review.

Due to the large structural diversity of naturally occurring alkaloids, we divided the sections attending to the number of cyclic units and according to the historical importance in the literature into seven families (see Scheme 1). Throughout the study, the alkaloids presented are natural products except otherwise indicated. 

### 4.1. Quinoline-Based Alkaloids

**Figure 2:**
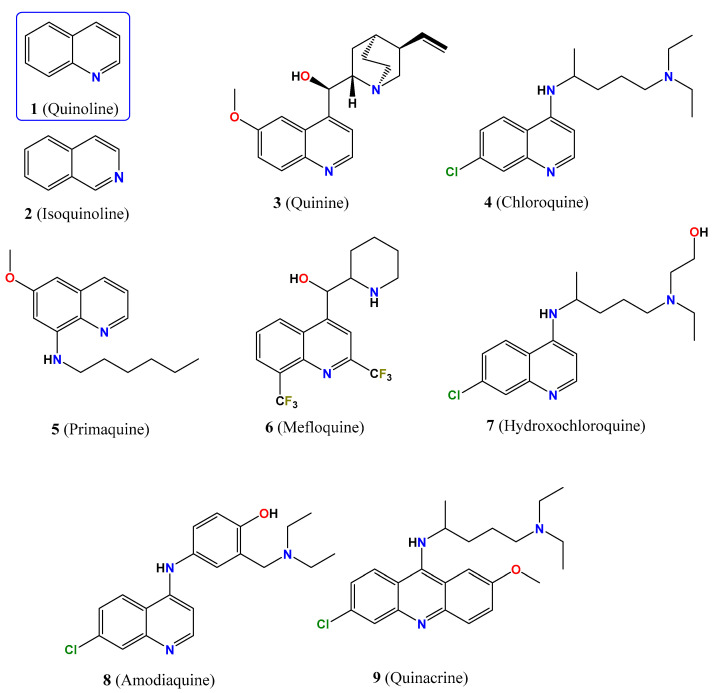
Quinoline-based alkaloids.

Quinoline (**1** in Figure 2) is an aromatic *N*-based bicyclic molecule acting as a weak ternary base and constituting one of the simplest and most important scaffolds of alkaloids. Quinolines are obtained from the bark of trees of the genus *Cinchona*, and they have been shown to exert a broad range of biological and therapeutic activities, including antibacterial, antifungal, and antimalarial activities. Quinoline derivatives show an absorption band ranging between 300 and 410 nm in the basic form, while the monoprotonated salts display a blue shift of the band. In addition, basic quinolines are nonemissive, while the protonated form exhibits a fluorescence emission band at 520 nm. Quinoline has a pK_a_ value of 4.85 and further modifications of the ring change this value [45,46].

Among quinoline-based alkaloids (**1**–**9**, Figure 2), quinine (**3**) is the most representative because it is the current ‘gold standard’ for the treatment of malaria. Quinine has three protonation species in aqueous solution, the di- and monoprotonated species, showing pK_a_ values of 4.2 and 8.3, and the neutral species, prevailing the monoprotonated species at pH 7. Alkaloid **3** presents the maximum absorption peak at 331 nm, and the fluorescence peak is centred at 367 nm at pH 7 (see Table 1), although the photophysical properties depend on the protonation state [47]. Under UV radiation (λ > 300 nm), **3** photooxidises several metabolites, including histidine, tryptophan, xanthine, and uric acid through a pathway mainly involving ^1^O_2_ with a maximum photoyield of 36%, calculated in D_2_O (Table 1). Other quinoline analogues **4**–**9** show low quantum yields for singlet oxygen generation, except **6** (38% in D_2_O), and all of them, except **5,** produce superoxide, hydroperoxyl, and peroxyl adducts in ethanol by means of electron paramagnetic resonance (EPR) spin-trapping assays.

In the early days, Tappeiner described the quinine photoactivity towards *Bacillus pyocyaneus* [48], as well as the photokilling process of the *Paramecium caudatum* [49,50], and *Amoeba proteus* protozoans under UV illumination [51]. Furthermore, UVA irradiation (14 J/cm^2^) sensitises quinine to photokill completely the yeast *Candida albicans* at 24 h [52]. Despite the importance in the pharmacology of quinoline alkaloids and the easy and numerous synthetic routes able to develop quinoline-based molecules, they are poorly demanded as PS because of the common absorption in the UV range of these compounds (≈110 references in 2021) [53], although the application of multiphoton technology to photoinactivation can reopen the interest for them nowadays.

### 4.2. Pterin-Like Alkaloids

**Figure 3:**
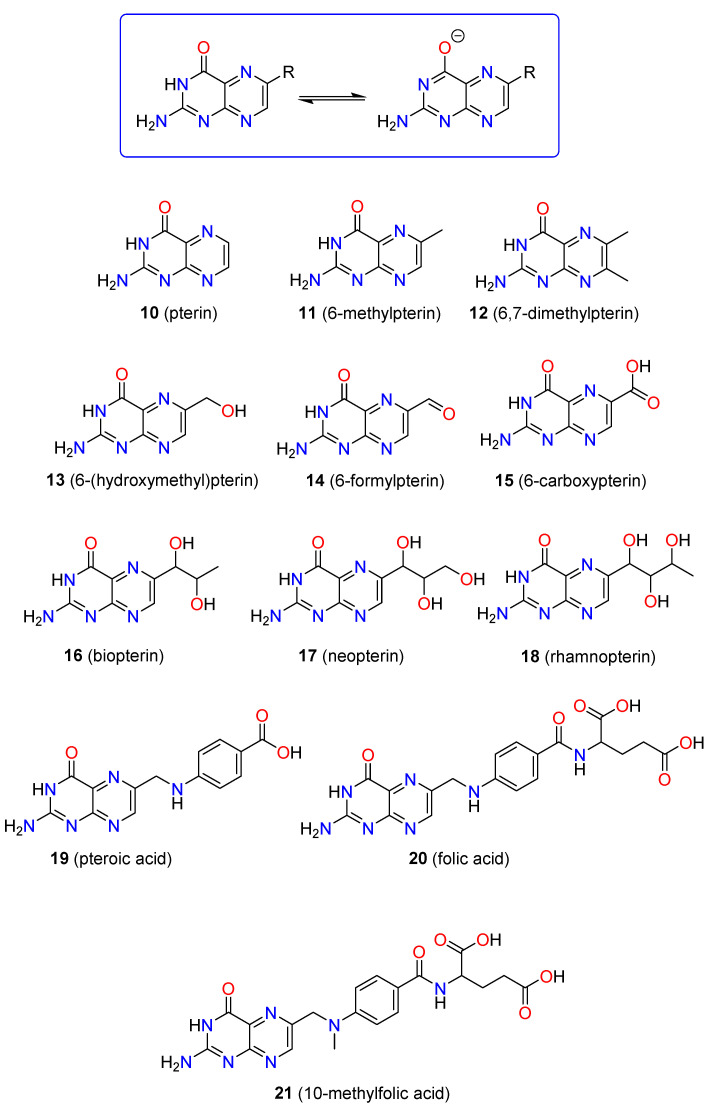
Pterin-based molecules.

The second class of alkaloids derives from the heterocyclic compound pterin (2-aminopteridin-4(1*H*)-one, **10** in Figure 3), in which a large series of derivatives results from the substitution of the six-ring position by different moieties (**10**–**21**, Figure 3). Pterins are obtained from butterflies, macrophages, and some insects, although several synthetic versions have appeared based on the naturally occurring ones. They behave as a monoprotic acid in an aqueous solution associated with the deprotonation of the amide moiety, and p*K*_a_ values range between 7.3 and 8.6 depending on the molecule; thus, the amide form prevails over the phenolate one at pH 7.4 (top panel in Figure 3). Nevertheless, pendant substituents can exhibit an additional protonation step [59]. Commonly, pterin derivatives show two absorption bands within the range 230–500 nm while exhibiting a fluorescence band at 450 nm. Pterins photosensitise throughout both mechanisms of action, type I and II, and are reported to oxidise a broad range of biomolecules such as nucleotides, amino acids, peptides, proteins, membranes, and DNA. The singlet oxygen yield greatly depends on the protonation state of the molecule, which generally increases under basic conditions (Table 2). Furthermore, the yield of singlet oxygen changes attending to the substitution of the heterocyclic ring, decreasing the quantum yield of singlet oxygen upon substitution of pterin scaffold [60]. Some investigations of the mechanism of action of pterin derivatives suggest oxidation of tryptophan and tyrosine to induce DNA crosslinks between DNA and proteins and/or photooxidative degradation of dGMP triggered by singlet oxygen and by an electron transfer [61,62,63]. Specifically, double-stranded DNA is damaged by pterin derivatives upon UVA irradiation, as it is sequence specific at the 5′ guanine of 5′-GG-3′ sequences [64]. The main drawback of pterins for photodynamic applications is the poor photostability either in the absence or in the presence of oxygen, causing the degradation of pterins under irradiation.

Carboxypterin (**15**) has been recently investigated for photoinactivation of *Staphylococcus aureus* (ATCC 25923) in both planktonic and biofilm cultures. Neither UVA nor solar irradiation reduced the bacterial growth, but the irradiation within the UVA range (λ_max_ = 350 nm, 17.3 J/cm^2^) for 2 h yielded a decrease in bacteria more than four orders of CFU for 0.1 and 0.2 µM treatments with **15** and killed all bacteria when using 0.4 µM. Sessile bacteria forming young biofilms were unable to survive on surfaces upon a combination of UVA irradiation (2 h) and 0.1 µM of **15**, although it became ineffective when reducing one order of magnitude the PS concentration. Interestingly, solar exposure was able to reduce three log10-units when using 100 µM of **15**, suggesting that sunlight is a potential light source for the bacteria inactivation. With regard to pre-existing biofilms, sessile cells decreased by more than three orders of magnitude upon UVA irradiation and treatment with **15** at 50 µM, indicative of the more resistant bacteria when forming biofilms [65].

**Figure 4:**
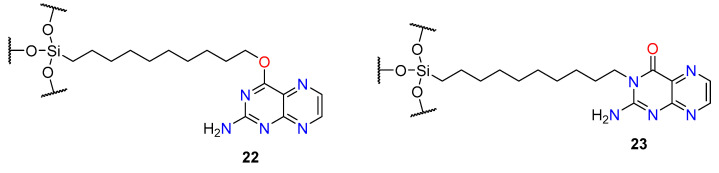
Alkyl substituted pterins.

*N* and *O* undecyl substituted pterins (**22**–**23**, Figure 4) have recently been described for the photodynamic inactivation of *S. aureus* (ATCC 25923) when immobilised on silicon substrates. UVA irradiation (λ_max_ = 365 nm, 20 min at 30 °C) achieves killing bacteria by 84%, in comparison with silicon substrate controls, indicating that pterin substitution induces a stronger photoinactivation of bacteria [66].

It is worth noting that some pterin derivatives act as light-harvesting antennas of the DNA photolyases upon UVA irradiation instead of generating ROS, which can be applied to target the DNA repair mechanism [59].

### 4.3. Carbazole Molecules

**Figure 5:**
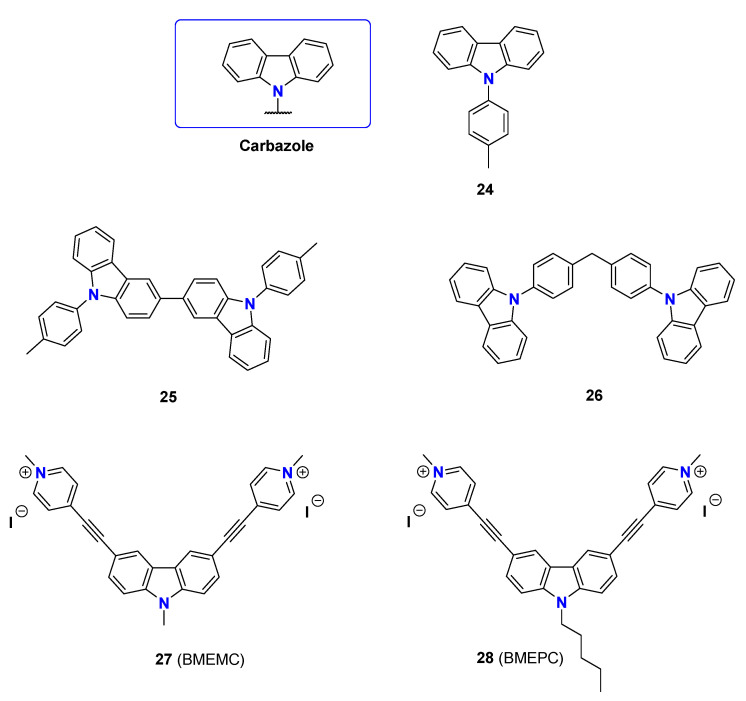
Carbazole-based photosensitisers.

Some alkaloids are characterised by a tricyclic scaffold consisting of a central pyrrole heterocycle fused to two benzene moieties termed carbazoles (**24**–**28**, Figure 5). They show interesting electronic and photophysical properties which have been pursued in energy transfer, materials, and technological applications. Moreover, natural and synthetic carbazoles have been used as therapeutic agents for microbial, protozoal, and insecticidal treatments, as well as in anti-inflammatory, antioxidative, and anti-HIV regimes [70]. Nevertheless, carbazoles have been poorly studied as photosensitisers for either microbial or other light activation regimes.

Recent insights into the singlet oxygen generation of synthetic *N*-phenyl carbazoles have shed light on their high sensitising capacity, ranging between 0.29 and 0.33, and their adequate photophysical properties for a two-photon absorption strategy. These molecules present several absorption bands, in which the most intense band is centred at 340 nm and an emission fluorescence band between 340 and 450 nm [71]. 

Two synthetic derivatives of carbazole showing 3,6-substitution have arisen as potent photosensitisers (**27**, BMEMC and **28**, BMEPC), with a *C*_2V_ symmetric structure and an acceptor–π–donor–π–acceptor configuration (acceptor/donor indicate π acceptor and π donor moieties, while π indicate π linkages between acceptor/donor moieties) [72,73]. Both ligands have two absorption bands at 330 and 420 nm, as well as an emission band centred at 576–590 nm. The planar V-shape structure allows calf thymus DNA to bind with moderate affinity (K_b_ = 2.9 × 10^−5^ and 3.3 × 10^−5^ M^−1^, respectively). Interestingly, they can photocleavage pBR322 DNA in a two-photon process upon light irradiation using an 800 nm laser pulse (12 fs, 1 kHz) in aerobic and anaerobic conditions, indicating a type I mechanism of action. EPR studies have assigned aminyl radicals as the radicals generated during the irradiation [74].

The carbazole-based molecules **27**–**28** showed antibacterial activity towards *E. coli* in the dark and under irradiation with laser at 442 nm (20 mW/cm^2^), which is the largest inhibitory zone for illuminated samples. Molecule **28** shows MIC values ranged between 6.9 and 13.8 µM in the dark and values between 3.5 and 6.9 µM under irradiation. In the dark, reaching MIC values within the 187.4–225 µM range were obtained for **27,** while 9.4–18.8 µM values were obtained under irradiation. The different activities are associated with the higher affinity of the longer alkyl chain to the cellular structure which yields a large accumulation of the molecules. In addition, the authors propose nitride radical formation upon light irradiation using EPR measurements and 5,5-dimethyl-1-pyrroline-*N*-oxide (DMPO) as the spin-trapping agent [75].

### 4.4. β-Carbolines Molecules

**Figure 6:**
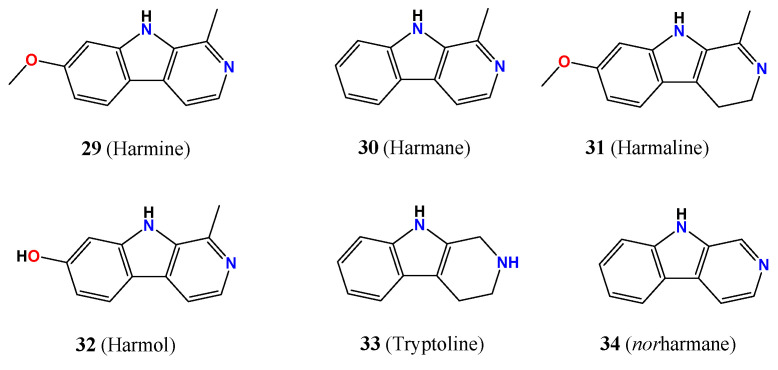
Carboline molecules.

*β*-Carbolines are an alkaloid family containing the 9*H*-pyrido[3,4-*b*]indole scaffold (**29**–**34**, Figure 6), among which harmine (**29**, 7-methoxy-1-methyl-9*H*-pyrido[3,4-*b*]indole) was the first to be isolated and the most representative molecule [13,76,77]. From its discovery, hundreds of *β*-carbolines have been reported from plants, fungi, and bacteria, besides being widely found in human and animal tissues such as eyes and skin.

A common photophysical property of β-carbolines is the moderate fluorescence and singlet oxygen quantum yields (Φ*_F_* and Φ_Δ_*,* respectively in Table 3). *β*-Carbolines show several protonation steps in aqueous solution, which change the photophysical properties and thus the capacity of being sensitised. Harmol (**32**, 1-methyl-9*H*-pyrido[3,4-*b*]indol-7-ol) presents two protonation steps with p*K*_a_ values of 7.8 and 9.6, associated with the protonation of the pyridinic nitrogen and the deprotonation of the hydroxy substituent, respectively. The UV–Vis spectrum of **32** shows the largest redshift in the pH range between both p*K*_a_ values (*ca.* 410 nm) [78]. Strikingly, **32** inhibited fungal growth of *Penicillium digitatum* and *Botrytis cinerea* in the dark only in acidic conditions (pH < 5), yielding considerable cellular damage. Under UVA photoactivation (30 min) and in acidic conditions, **32** required half of the dose to reach the same inhibitory effect for *B. cinerea* without photoactivation. In contrast, *P. digitatum* growth was not significantly altered using photoactivation (1.15 and 0.5% viability for dark and irradiated fungi, respectively). In line with the inhibitory effect, **32** photoactivation did not increase ROS in *P. digitatum*, compared with treatment with **32** in the dark, whereas a six-fold increase in ROS was observed in *B. cinerea*. Mechanical studies indicated that both types of mechanisms are possible for damage generation.

Harmine (**29**) has been photoactivated using UVA irradiation in cancer cells, generating DNA photooxidation (purine oxidation and cyclobutane pyrimidine dimers) [79]. Both types of mechanisms are plausible in cancer cells, and lysosome localisation is assigned from confocal microscopy.

*Nor*harmane (**34**) shows only a protonation step occurring at pH 7.2, assigned to the *β*-nitrogen protonation with a concomitant redshift of 50 nm. Upon UVA irradiation, **34** generates ROS and photooxidise AMP nucleotide through a type I mechanism via H_2_O_2_ [80].

### 4.5. Aporphine Alkaloids

**Figure 7:**
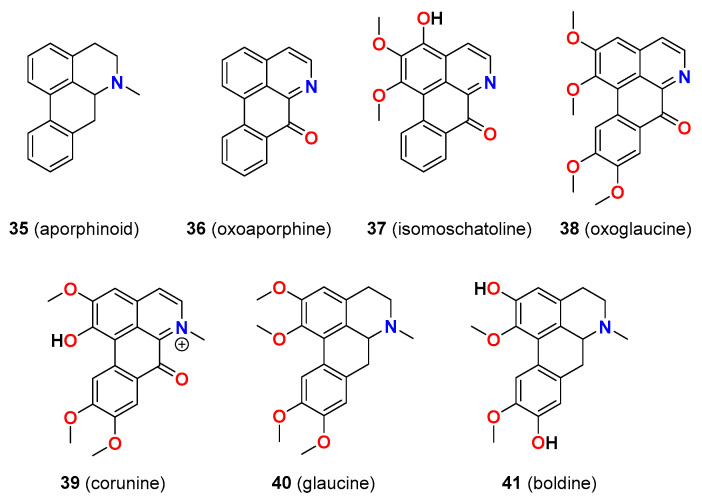
Aporphine-like alkaloids.

Annonaceae is a large family of aromatic trees, shrubs, or climbers, which occur in tropical and subtropical regions. They originate from several interesting alkaloids with an isoquinolinic structure (benzylisoquinolines **35**–**41**, Figure 7) such as aporphines. They can act as photosensitisers or antioxidants depending on the structure, in particular the incorporation of hydroxyl groups in the isoquinolinic scaffold [86]. For instance, boldine (**41**) and glaucine (**40**) are efficient ROS quenchers while oxoglaucine (**38**) is a potent PS with a high photoyield of ^1^O_2_ (100%, Table 4) in apolar solvents [87], although it has not been investigated for aPDT.

Isomoschatoline **37** shows several absorption bands covering the optical window with maxima at 287, 320, 470, and 660 nm and an emission band at 560 nm (λ_exc_ = 420 nm, Table 4). In addition, **37** generates singlet oxygen, which was quantified using the decomposition of 1,3-DPBF (1,3-dimethylisobenzofuran) to afford a 40.2% reduction in absorption. A photoirradiation assay of *Staphylococcus epidermidis* (ATCC 12228) using **37** and ROS scavengers suggested that all scavengers improved the bacteria viability, indicating that both mechanisms I and II were involved in the antimicrobial effect. The irradiation (660 nm, 28 J/cm^2^, 2 min) of **37** showed a minimum of three-log growth reduction in bacteria *S. aureus* (ATCC 10538), *S. epidermidis* (ATCC 12228), *E. coli* (ATCC 10799), and yeasts *C. albicans* (ATCC 1023) and *C. dubliniensis* (ATCC157778). The growth reduction in Gram-positive bacteria and yeasts of **37** between nonirradiated and irradiated was found to be 42%, while *E. coli* presented 36%. Interestingly, energy density variation assays indicated a larger growth reduction at high energy doses of 56 J/cm^2^, while no differences were observed for higher doses [88].

### 4.6. Protoberberine-like Alkaloids

**Figure 8:**
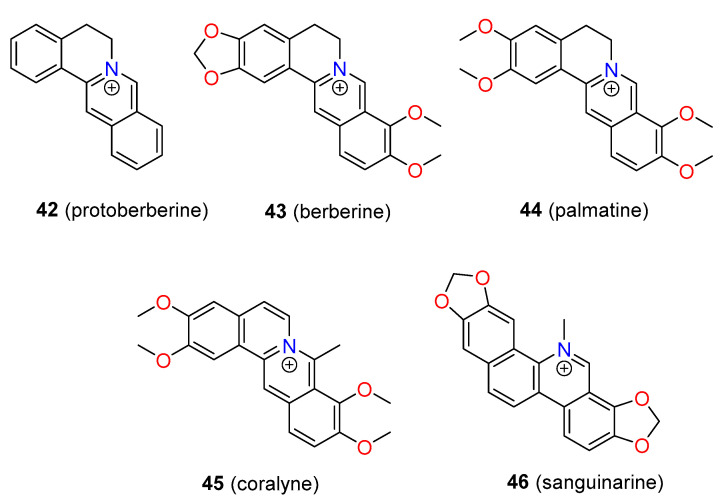
Protoberberine-like alkaloids.

A class of polycyclic alkaloids includes the isoquinolines described as protoberberines (**42**–**46**, Figure 8), which are isolated from *Berberis vulgaris*, *Sanguinaria canadensis*, or *Mahonia aquifolium*. In general, protoberberines present the absorption bands within the UVA region and some in the UVB and visible region. Berberine (**43**) and palmatine (**44**) have two absorption bands centred at 360 and 430 nm and fluorescence at 590 and 581 nm, respectively, in organic solvents. No fluorescence or weak fluorescence was observed for protoberberines in an aqueous solution because of the aggregation or change in polarity of the local microenvironment [89,90]. In organic solvents, **43** and **44** produce ^1^O_2_, while no production was observed in water (see Table 4). Further, **43** and sanguinarine (**46**) present photophysical changes across the pH range. Even being poorly emissive in aqueous solutions, **43** shows a light-up effect in organic solvents and generates different ^1^O_2_ amounts depending on the solvent (photoyield of 0.34 in dichloromethane) [91]. EPR trapping assays indicated the generation of hydroxyl radical upon UVB irradiation and phototoxicity and perinuclear localisation in HaCat cells, although photoinactivation of microbial cell studies has not been conducted yet [92].

### 4.7. Indigo-Like Alkaloids

**Figure 9:**
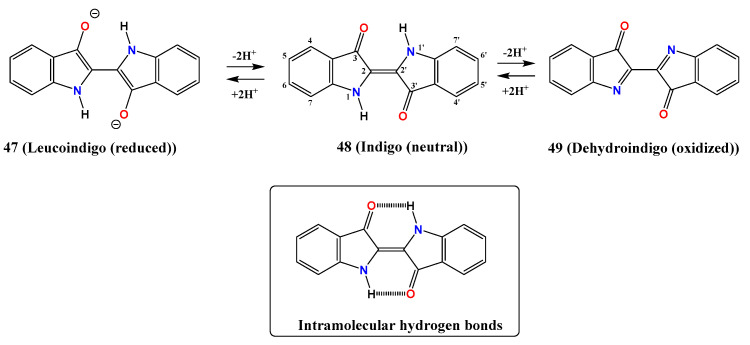
Indigo equilibria in solution.

**Figure 10:**
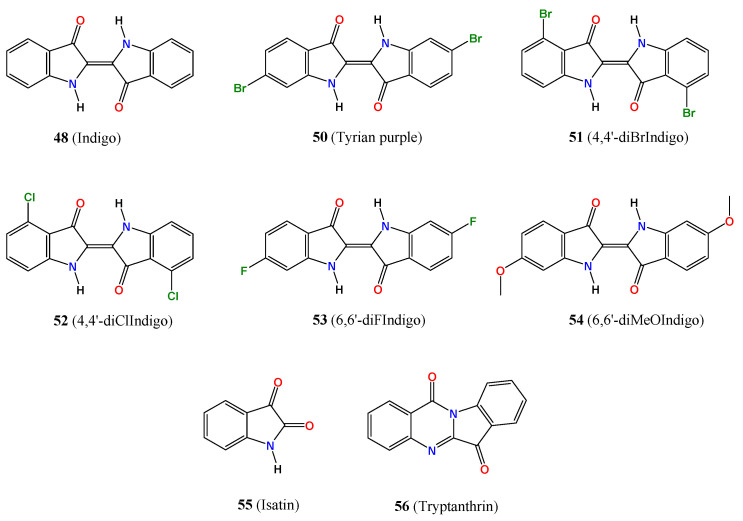
Indigo-like alkaloids.

Indigo-like alkaloids (Figure 9 and Figure 10) are one of the most popular and ancient families of molecules used for a variety of applications; indigo is a worldwide dye used today for colouring uniforms blue. Indigo and derivatives are obtained from the leaves of plants from the *Indigofera* genus, which is found mainly in Central and South America. Indigo presents three different forms—namely, (i) a keto form, (ii) a leuco form, and (iii) a dehydro form (see in Figure 9), the later ones resulting from the reduction and oxidation of the neutral keto form [93]. Seixas de Melo et al. have investigated the photophysical and photochemical properties of indigo derivatives (Table 4). Indigo-like molecules are insoluble in water and become water soluble upon reduction to leucoindigo. They are highly stable because of the intramolecular hydrogen bond formation between adjacent carbonyl and amine groups, locking the molecules into a trans-planar configuration and preventing them from any isomerisation.

As keto species, indigo shows a UV–Vis spectrum with a visible band centred at 610 nm and a fluorescence emission spectrum within the region 640–660 nm, while the leuco species display an absorption band centred at 440 nm and an emission band at 525 nm (Table 4). The S_1_→T_1_ process has high efficiency in the leuco species due to the proximity between both states and populates the triplet state which, in turn, is an alternative to the internal conversion and other efficient deactivation channels for the singlet excited state [94]. On the other hand, indigo-like molecules as keto species show highly efficient internal conversion S_1_→S_0_, while as a leuco species, fluorescence emission and the triplet state is populated deactivating the excited state. These photophysical properties envisaged adequate features for photosensitising leuco species but not keto ones.

The quantum yield of singlet oxygen formation, based on the characteristic phosphorescence signal of the single oxygen at 1270 nm in dimethylformamide, is fairly low (*ϕ*_Δ_ < 0.01) for indigo. Synthetic derivatives of keto-like indigo at 4,4′ positions by chlorine and bromide increase the *ϕ*_Δ_, while 6,6′ derivatisation with bromide and methoxy decreases the quantum yield, and only fluorine groups enhance the oxygen sensitisation (see Table 4). In general, indigo and derivatives have very low sensitised singlet oxygen yields, indicating inefficient singlet oxygen sensitisation because of the very low S_1_→T_1_ intersystem crossing [95].

The higher reduction of indigo forms the dehydrogenated species named dihydroindigo, which shows an absorption band in the visible region centred at 455 nm in aprotic solvents and 400 nm in protic ones. Interestingly, the quantum yields are high (*ϕ*_Δ_ < 0.6 in toluene, *ϕ*_Δ_ < 0.83 in benzene, and *ϕ*_Δ_ < 0.15 in methanol) for the dihydroindigo, indicating an efficient energy transfer between the triplet state to molecular oxygen [96].

Andreazza et al. described the photophysical parameters of *Indigo truxillensis* extracts within the potential applications for aPDT with strong visible bands ranging between 600 and 800 nm, centred at 660 nm, and fluorescence emission around 670 nm upon excitation at 620 nm. The assessment of the microorganism viability under irradiation with different ROS scavengers and the single oxygen quantification using the 1,3-DPBF assay suggested a type I mechanism for indigo photodynamic action [97]. The bacteria and yeast strains (*S. aureus* ATCC 14458; *S. epidermidis* ATCC 12228; *E. coli* ATCC 10799; *P. vulgaris* field strain; *C. albicans* ATCC 1023 and ATCC 10231; *C. dubliniensis* ATCC 778157 and ATCC 777) showed a significant decrease in the viabilities upon light irradiation (>80% in the number of CFU/mL) in comparison with nonirradiated microorganisms. A general trend was observed for indigo, denoting a lower susceptibility in Gram-negative bacteria than Gram-positive. To increase the permeability of the outer membrane of Gram-negative bacteria to indigo, CaCl_2_ or MgCl_2_ were added to the treatment of *E. coli* ATCC 10799 before irradiation. An increase in bacterial susceptibility was shown for both salts, higher for CaCl_2_. Among yeast strains, *C. albicans* showed the largest photodynamic inactivation, although less effective than for bacteria.

Other indigo-like molecules—isatin (**55**) and tryptanthrin (**56**)—were evaluated against bacteria and fungi, resulting in a weak inhibitory effect of **55,** while **56** strongly inhibited the growth of *S. epidermis* (ATCC 12228) and *S. aureus* (ATCC 6538) and a weak effect on methicillin-resistant *Staphylococcus aureus* (MRSA, ATCC 43000) [103]. In addition, **56** has been assayed with moderate effect on *B. subtilis* [104] and *Mycobacterium tuberculosis* [105]. All these molecules can potentially exhibit higher activity when irradiated acting through a native mechanism in tandem with the photoactivation.

Interestingly, the modern chemical industry has increased the production of indigo because of the high demand for textiles which opens new opportunities to explore the indigo-like molecules in a low-cost format. The substitution and the orientation of the electron donor (-NH) and acceptor (-C=O) tightly regulate the photophysical and photochemistry of these molecules which, in turn, can evolve new and most potent photosensitisers.

## 5. Conclusions

Alkaloids are an important class of natural products with biomedical applications, and the combination of their photoactivation with light paves new avenues to inactivate microorganisms. Herein, we compiled the studies dealing with the application of aPDT using alkaloids and detailed the main conclusions. We gathered the key photophysical properties such as emission and absorption maxima wavelengths, quantum singlet oxygen yield, or the lifetime to apply and select alkaloids as PS. Of utmost importance is the easy obtention of these molecules, which can be applied in a low-cost format to tackle microbial infections as an alternative approach to antibiotic treatments. Among the alkaloids reviewed, aporphines and protoberberines show the largest redshift of the absorption and emission bands, and thus, they can be potentially more interesting for aPDT. Nevertheless, multiphoton technologies able to excite high energy wavelengths can open new venues to use all alkaloids and photosensitise them into the UV region. We expect this review to be used as a guideline to obtain the main features for the application of alkaloids in aPDT.

## Figures and Tables

**Table 1:** Photophysical properties of quinoline-based alkaloids.

Compound	λ_abs_ (nm)	λ_em_ (nm)	Φ*_F_*	Φ_Δ_	τ*_F_* (ns)	References
**3**	331 ^a^	367 ^a^	0.55 ^b^	0.36 ^c^	<10^−3 d^	[48,51,54]
**4**	373 ^a^	380 ^a^	5 × 10^−3 a^	<10^−2^	0.31, 0.69 ^a^	[47,50,55,56]
**5**	259 ^a^, 350 ^a^, 266 ^c^	522 ^a^	3.5 × 10^−3 a^	<10^−2^	0.06, 0.94 ^a^	[47,54]
**6**	284 ^a^	377 ^a^		0.38 ^c^	<10^−3 d^	[49,57]
**8**	210, 256, 341 ^a^	480 ^a^, 410 ^e^	1.5 × 10^−2 e^, ≤10^−4 a^	10^−2^–10^−4^	<0.2 ^a^	[47,50,54,58]

^a^: in PBS, pH = 7.2; ^b^: in 0.1 N H_2_SO_4_; ^c:^ in D_2_O; ^d^: in EtOH; ^e^: in MeCN. λ_exc_ = λ_abs_. Data obtained from references [47,48,49,50,51,54,55,56,57,58].

**Table 2:** Photophysical properties of pterin-based alkaloids.

Compound	pH	λ_abs_ (nm)	λ_em_ (nm) ^a^	Φ*_F_* ^a^	Φ_Δ_	τ_F_ (ns) ^a^	References
**10**	4.9–5.5	270, 340	439	0.33	0.18	7.6	[62,63,64]
	10–10.5	252, 358	456	0.27	0.3	5.0	
**11**	4.9–5.5	271, 347	448	0.61	0.10	13.3	[64]
	10–10.5	252, 363	460		0.14	11.2	
**12**	4.9–5.5	273, 344	433	0.85	0.04	13.5	[59,63,65,67,68]
	10–10.5	250, 358	445	0.84	0.1	11.6	
**13**	4.9–5.5	275, 345	449	0.53	0.15	11.0	[59,63,64,68,69]
	10–10.5	254, 364	457	0.46	0.21	8.4	
**14**	4.9–5.5	276, 316	446	0.1	0.45	7.9	[59,62,63,64,67,68,69]
	10–10.5	280, 370	454	0.02	0.47	2.2	
**15**	4.9–5.5	286, 346	439	0.28	0.27	5.8	[59,62,63,65,67,68,69]
	10–10.5	264, 364	451	0.18	0.37	4.1	
**16**	4.9–5.5	274, 346	441	0.36	0.34	9.1	[59,63,64,65,68,69]
	10–10.5	254, 363	455	0.29	0.4	7.6	
**17**	4.9–5.5	274, 346	440	0.38	0.23	8.9	[59,63,64,65,68,69]
	10–10.5	254, 363	454	0.31	0.34	7.4	
**18**	4.9–5.5	273, 344	441	0.47	0.13	10.7	[59,63,65,68,69]
	10–10.5	254, 363	455	0.40	0.16	7.5	
**19**	4.9–5.5	279, 347	450	6.1 × 10^−3^	<0.02	3.9	[59,63,65,68,69]
	10–10.5	257, 366, 277	460	7.9 × 10^−3^			
**20**	4.9–5.5	285, 354	445	<0.005	<0.02	7.0	[62,63,64,65]
	10–10.5	255, 365, 285	455			3.5	
**22**		234, 263, 354 ^b^	428 ^b^	0.012 ^b^	1.2 ^b^	0.5 ^b^	[60]
**23**		240, 278, 348 ^b^	428 ^b^	0.043 ^b^	0.8 ^b^	0.36 ^b^	[60]

Data obtained in H_2_O at the pH indicated except otherwise indicated. ^a^: λ_exc_ = 350 nm. ^b^: in MeCN. The excitation wavelength used was data obtained from references [59,60,62,63,64,65,67,68,69].

**Table 3:** Photophysical properties of carbazoles and carbolines.

Compound	λ_abs_ (nm)	λ_em_ (nm)	Φ*_F_*	Φ_Δ_	τ_F_ (ns)	References
**24**	293, 329, 342 ^a^	363 ^a^ (λ_exc_ = 340 nm)	0.34 ^a^	0.33 ^a^		[71]
**25**	304, 342, 356 ^a^	340 ^a^ (λ_exc_ = 340 nm)	0.12 ^a^	0.29 ^a^		[71]
**26**	293, 329, 342 ^a^	363 ^a^ (λ_exc_ = 340 nm)	0.31 ^a^	0.30 ^a^		[71]
**27**	330, 420 ^b,c^	592 ^b,c^ (λ_exc_ = 415 nm)	<0.001 ^b,c^			[74]
**28**	330, 420 ^b,c^	576 ^b,c^ (λ_exc_ = 415 nm)	<0.001 ^b,c^			[74]
**29**	250, 300, 370 ^d^	430 ^d^	0.42 ^d^	0.35 ^d^	5.5 ^d^	[81,82,83]
**30**	289, 299, 349 ^d^	440 ^d^	0.67 ^d^	0.58 ^d^	8.5 ^d^	[81,83,84,85]
**31**	375 ^d^	490 ^d^	0.44 ^d^		4.0 ^d^	[81,83,85]
**32**	330 ^b^	435 ^b^	0.39 ^b^		5.1 ^b^	[78,83,85]
**34**	250, 305, 380 ^b^ (pH = 4.8)	469 ^b^	0.57 ^b^	0.40 ^d^	10.6 ^b^	[80,81,83,84,85]
	235, 280, 350 ^b^ (pH = 10)	469 ^b^	0.57 ^b^	0.40 ^d^	10.6 ^b^	

^a^: in DMF, ^b^: in H_2_O, ^c^: in DMSO, ^d^: in MeCN, [71,74,78,80,81,82,83,84,85].

**Table 4:** Photophysical properties of aporphines, protoberberines, and indigos.

Compound	λ_abs_ (nm)	λ_em_ (nm)	Φ*_F_*	Φ_Δ_	τ_F_ (ns)	References
**37**	287, 320, 470, 660 ^a^	540 ^a^ (λ_exc_ = 470)				[87]
**38**	242, 272 ^b^	510 ^b^	0.002 ^b^	1 ^c^	6.7 ^b^, <0.1 ^b^	[86,88,98]
**39**				0.025 ^d^		[88]
**43**	421 ^b^	555 ^b^, 524 ^c^	0.02 ^d^, 0.07 ^e^	0.00045 ^b^, 0.34 ^f^, 0.04 ^d^		[88,91]
**44**	350, 430 ^b^	541 ^f^, 581 ^d^, 519 ^e^	0.37 ^f^, 0.01 ^d^, 0.24 ^e^	0.20 ^f^, 0.11 ^d^, 0.04 ^e^		[91,99]
**45**	310, 425 ^b^	525 ^b^	0.05 ^b^	0.7 ^c^		[100,101]
**46**	327 ^f^		0.004 ^f^	0.005 ^f^	2.38 ^f^	[99,102]
**48**	415 ^e^, 442 ^g^	500 ^e^, 523 ^g^	0.46 ^e^, 0.348 ^g^	0.00117 ^g^	3.33 ^e^, 0.315 ^g^	[92,95]
**50**	430,442 ^g^	505 ^e^, 526 ^g^	0.22 ^e^, 0.225 ^g^	0.0005 ^g^	3.77 ^e^, 3.77 ^g^	[92,93,95]
**51**	430 ^g^	505 ^g^	0.38 ^g^	0.002 ^g^	2.89 ^g^	[92,95]
**52**	430 ^g^	503 ^g^	0.22 ^g^	0.001 ^g^	3.2 ^g^	[92,95]
**53**	412 ^g^	518 ^g^	0.04 ^g^	0.001 ^g^	0.55 ^g^	[92,95]
**54**	413 ^g^	515 ^g^	0.05 ^g^	0.0005 ^g^	0.55 ^g^	[92,95]

^a^: in DMSO, ^b^: in H_2_O, ^c^: in benzene, ^d^: in MeCN, ^e^: in dioxane, ^f^: in dichloromethane, ^g^: in DMF. Data obtained in references [86,87,88,91,92,93,95,99,100,101,102].

## Data Availability

Not applicable.
